# Efficient pump-probe sampling with a single-cavity dual-comb laser: Application in ultrafast photoacoustics

**DOI:** 10.1016/j.pacs.2022.100439

**Published:** 2022-12-13

**Authors:** J. Pupeikis, W. Hu, B. Willenberg, M. Mehendale, G.A. Antonelli, C.R. Phillips, U. Keller

**Affiliations:** a ETH Zurich, Auguste-Piccard-Hof 1, Zurich 8093, Switzerland; b Onto Innovation Inc., 16 Jonspin Road, Wilmington, MA 01887, USA

**Keywords:** Ultrafast pump-probe, Picosecond ultrasonics, Pump-probe microscopy, Time domain thermoreflectance, TDTR, Time-resolved Brillouin oscillations, Thin-film layer thickness measurements, Non-destructive testing

## Abstract

Ultrafast pump-probe measurements are used to characterize various samples, such as biological cells, bulk, and thin-film structures. However, typical implementations of the pump-probe apparatus are either slow or complex and costly hindering wide deployment. Here we combine a single-cavity dual-comb laser with a simple experimental setup to obtain pump-probe measurements with ultra-high sensitivity, fast acquisition, and high timing precision over long optical delay scan ranges of 12.5 ns that would correspond to a mechanical delay of about 3.75 m. We employ digital signal balancing to obtain shot-noise-limited detection compatible with pump-probe microscopy deployment. Here we demonstrate ultrafast photoacoustics for thin-film sample characterization. We measured a tungsten layer thickness of (700 ± 4) Å with shot-noise-limited detection. Such single-cavity dual-comb lasers can be used for any pump-probe measurements and are especially well-suited for ultrafast photoacoustic studies such as involving ultrasonic echoes, Brillouin oscillations, surface acoustic waves and thermal dynamics.

## 1 Introduction

Ultrafast pump-probe measurements enable access to information which is not available using linear reflectivity with the probe alone [1]. For instance, in the case of opaque thin films, a picosecond ultrasonics technique was developed in the 1980s [2]. An incident ultrashort pump pulse creates local heating which initiates an acoustic wave packet of very high frequencies in the GHz to THz range. As the wave packet propagates through the sample it gets reflected at various interfaces. The returning signals create a reflectivity change that can be sampled with a time-delayed probe beam. This measurement technique is successfully deployed in the microchip inspection industry [3]. The latest advances in ultrafast photoacoustics enable label-free imaging of biological cells using mechanical properties as the contrast agent [4], [5], [6]. The mentioned properties are typically obtained by observing time-resolved Brillouin oscillations, which are in the GHz frequency range. Another ultrafast laser-based measurement application is time-domain thermoreflectance (TDTR) which is used to measure the thermal properties of materials and study the transport processes of energy carriers [7], typically in the sub-GHz frequency range.

A common feature of these mentioned applications is that they require measurement of long optical pump-probe delays, sometimes up to 10 ns which is equivalent to 3-meter light propagation in free space. For example, a long scan range in picosecond ultrasonics enables the study of complex thin-film stacks with several tens of micrometer total thickness, as encountered in modern semiconductor microchips. Furthermore, long pump-probe delays are needed to resolve surface acoustic waves and thermal dynamics. Unfortunately, scanning over such long physical distance with a mechanical delay line is slow, susceptible to systematic errors in the measurement due to beam deflection or divergence, and implies a complex optomechanical system [8]. The system complexity is further increased since the slow optical delay scan speed requires lock-in detection of the signal in order to shift the data acquisition to higher frequencies where probe relative intensity noise is smaller.

Alternatively, long optical delay scans can be obtained by using an equivalent time sampling (ETS) concept [9] demonstrated with electro-optic sampling using a microwave pump signal and a probe laser pulse train with different repetition rates. This was also implemented for optical spectroscopy applications using slightly different repetition rates for the pump and probe lasers [10]. This type of sampling with two different optical pump and optical probe repetition rates became also known as asynchronous optical sampling (ASOPS) and is now commonly used for ultrafast photoacoustic studies, for example, for scans over 100 ps [11], 1 ns [12], 10 ns [13] and 21 ns [14]. We would like to suggest using ETS instead of ASOPS because the pump and probe pulse trains are still synchronized with each other at a small difference in pulse repetition rates. The scan range is given by the inverse of the pump repetition rate *f*_pump_ and the scan speed is determined by the pump and probe repetition rate difference Δ*f*=*f*_pump_-*f*_probe_. Thus, the measurement time (i.e. laboratory time) is related to the pump-probe time via the ratio of *f*_pump_/Δ*f*.

In practice, ETS is implemented with two separate ultrafast lasers which are synchronized via high-frequency phase-locked loops and high-bandwidth feedback electronics. This, however, leads to expensive and bulky experimental setups hindering wide adoption. Achievable synchronization performance from such systems depends on several factors such as electronic feedback bandwidth with typically reported period jitter being 160 fs over 10 ns scan range [13] and 503 fs over 21 ns [14]. The ETS time axis stability then can be estimated by the ratio of these two quantities indicating ∼10^−5^ typical stability on the time axis.

Dual-comb modelocking in a single laser cavity, such as in fiber [15] or solid state [16], [17] architectures, has emerged as a promising alternative to stabilizing two separate lasers. Because the two pulse trains share the same optical cavity, they experience the same perturbations and thus the relative timing jitter is low without any feedback. The solid-state architecture is especially appealing for dual-comb lasers, firstly, because ultralow noise performance can be achieved due to low intracavity nonlinearity and losses [18], [19]. Secondly, femtosecond pulses with high pulse energies can be obtained without the use of chirped-pulse amplification [19], [20], [21]. Finally, crosstalk between the two pulse trains inside the same cavity can be negligible as opposed to single-cavity fiber-based designs where pulse collisions are unavoidable [22].

We have lately demonstrated a polarization-multiplexed high-power solid-state single-cavity dual-comb laser and applied it for picosecond ultrasonics, time-resolved Brillouin spectroscopy [20] and semiconductor saturable absorber mirror characterization [23]. In this report we expand on this approach by using a higher performance, spatially-multiplexed single-cavity dual-comb oscillator delivering up to 2.4 W of average power per comb with sub-140-fs pulses. The relative time axis stability was characterized to be 2·10^−7^, corresponding to just a few femtosecond period jitter for the complete 12.5 ns scan range as reported in [19]. The new multiplexing approach allows to control the repetition rate difference with a low-bandwidth frequency lock and thus to cancel any relative frequency drifts occurring in the setup, making the stability of the time axis to be excellent regardless of changes in the environment. Furthermore, our previously demonstrated ETS pump-probe data acquisition methods had some important trade-offs. Firstly, in case of balanced detection, only low optical power was accessible before photodiode saturation, thus increasing the shot and thermal noise contributions significantly. Secondly, the analog signal balancing, for the partially shot or thermal-noise limited signal, was increasing the noise floor due to the uncorrelated nature of the noise. Finally, high-power detection using a single high-power photodiode was limited in the low frequency band of the detection due to a bias-tee in the circuit thus effectively cutting the low frequency signals away.

In this report, we adopt the new spatially-multiplexed single-cavity dual-comb laser for ultrafast photoacoustic measurements, and we demonstrate that high-performance signal acquisition can be obtained with a low shot-noise contribution. ETS is a powerful measurement method because it allows sampling over long optical delay range with femtosecond precision at km/s equivalent optical delay scan speeds, which are by far not obtainable via mechanical means. However, a careful measurement system design and noise considerations are needed to benefit from such fast scan speeds. Here we perform detailed noise analysis for the ETS case and identify where the signal becomes shot-noise limited. In addition, we explore how a shot-noise-limited ETS measurement noise floor scales depending on acquisition parameters. Finally, we demonstrate a new signal balancing scheme which is compatible with setups for pump-probe microscopy applications, requiring rebalancing at Δ*f* rate. Our results demonstrate a high-performance, yet low-complexity pump-probe apparatus ideally suited for ultrafast photoacoustics.

## 2 Experimental setup and signal processing

**Fig. 1:**
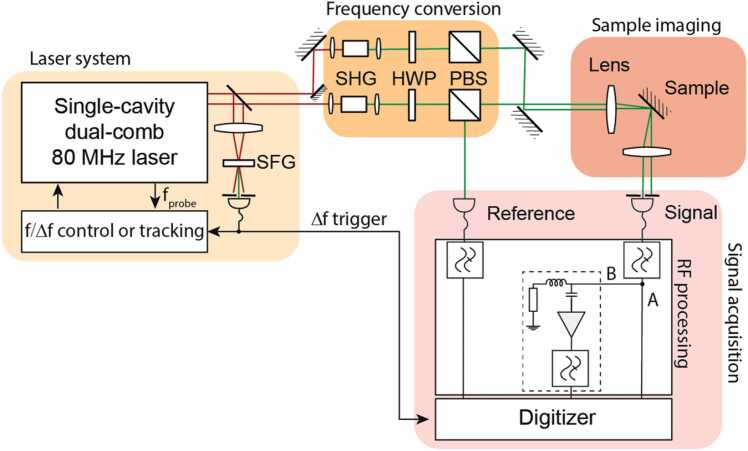
Experimental setup. A trigger signal is generated by sum-frequency generation (SFG) between the two output combs. Each of the combs goes through separate second harmonic generation (SHG) stages and the output powers are controlled by a half-wave plate (HWP) and polarizing beam splitter (PBS) pairs. The radio-frequency (RF) processing for the signal uses one of two schemes: either A or B. Indicated low-pass filters are 48 MHz (BLP-50 +, Mini-Circuits) each. The A scheme consists only of low-pass filters. The B scheme consists of a bias-T (BT45R, SHF Communication Technologies AG) to separate the AC and DC parts of the signal. The DC part is terminated with a 50-Ω resistor. The AC part is amplified with a voltage amplifier (DUPVA-1–70, Femto Messtechnik GmbH).

Our pump-probe setup is shown in Fig. 1 where the shading highlights the different conceptual elements. The setup is powered by a single-cavity dual-comb laser operating at 80 MHz and delivering two pulse trains with tunable repetition rate difference, based on a similar configuration as presented in detail in [19]. The laser is operated at 500 Hz repetition rate difference with sub-140 fs pulse duration output. The pulses reaching the experimental setup are slightly stretched due to multiple polarizing beam splitting cubes in the path.

A small part of each beam is split off to perform cross-correlation of the two output combs in a β-BaB_2_O_4_ (BBO) crystal phase-matched for sum-frequency generation. The signal is detected with an amplified photodiode (PDA55, Thorlabs Inc.). The output from this photodiode is used to trigger the signal acquisition at the moment the pulses overlap in time. We also use the generated trigger signal to track and, if necessary, control the repetition rate difference Δ*f*. In our earlier work, we have shown that precise pump-probe measurements can be performed with a free-running laser configuration [20], [23]. However, it is convenient to fix the repetition rate difference to an exact value so that environmental changes would not impact the measurements. Therefore, we control the repetition rate difference Δ*f* by adjusting the intra-cavity multiplexing element (i.e. the biprism). Specifically, to stabilize Δ*f*, the cross-correlation signal is sent to a customized frequency counter which infers Δ*f* via the delay between peaks in the cross-correlation signal. The inferred value of Δ*f* is read out digitally and used in a slow feedback loop (up to Δ*f* update rate) to adjust the position of the intracavity biprism with a piezoelectric actuator. In [19], we confirmed the successful operation of this approach by an out-of-loop frequency counter measurement. Thus, in these measurements, we controlled Δ*f* with a slow frequency lock and simultaneously recorded the pump repetition rate during the data acquisition.

Each comb is directed into the second harmonic generation (SHG) stages based on 5-mm-long lithium triborate (LBO) crystals where 550 mW of 525 nm light is generated. We use half-wave plate (HWP) and polarizing beam splitter (PBS) pairs to fine-control the optical powers. For the experiments, we have used a 25 mm focal length aspheric lens to reach 15×20 μm^2^ pump and 14×18 μm^2^ probe beam diameters (1/e^2^) when accounting for beam projection due to 45° angle of incidence (AOI) on the sample. We maintain a small noncollinearity in the plane of incidence between the pump and probe so that the probe can be effectively separated for detection. For the presented experiment the pump and probe polarizations were p and s, respectively. The chosen geometry was convenient for extracting the probe light without any additional losses or the need for special optical components. Furthermore, this configuration allows for ellipsometry measurements in the same setup [24], which in combination with the photoacoustic measurement can be used to determine the speed of sound inside the sample.

The signal and the reference beams are detected with two separate reverse-biased 0.8-mm^2^ InGaAs photodiodes with 70 MHz detection bandwidth. The optical power of the signal and reference beams on the photodetectors is typically set to 17 mW of SHG. We target to detect signals well below the noise floor of our digitizer, ideally down to 10^−5^ relative modulation of the DC component per single trace. We solve this challenge by splitting our signal acquisition into two schemes: A and B as shown in Fig. 1. The A scheme is used for reference and signal detection. We digitize the low-pass filtered signal and reference simultaneously with a data acquisition card (PCI-5122, National Instruments) at a 100 MS/s sampling rate and 50-Ω DC coupling. This signal will be limited by the data acquisition card noise, in ∼10^−4^ relative sensitivity range. This is enough to capture relatively low-frequency components of the signal, but it is not sufficient to resolve higher-frequency noise components (i.e., >100 kHz). Thus, to enhance the detection sensitivity for the high-frequency components of the signal, we use detection scheme B as shown in Fig. 1. We then merge data from the two schemes to arrive at the final measurement using a procedure discussed in the next section. Because our data acquisition card supported only two input channels, we had to first record the A scheme for the signal and the reference and then the scheme B for the high-sensitivity measurement of the signal. In an optimized implementation, one would use a four-channel acquisition card which would allow using the B scheme for the signal and reference simultaneously. To further reduce the noise, we configure the data acquisition card to simultaneously average the traces on different channels following the timing signal from the trigger.

The described acquisition yields three averaged voltage time traces: $V_{\mathrm{reference}}^{A}$, $V_{\mathrm{signal}}^{A}$, and $V_{signal}^{B}$, where A and B indicate the measurement scheme. In the next section, the digital processing steps for the obtained voltage traces are described which yield the measured relative reflectivity quantity Δ*R*/*R*.

## 3 Digital processing and measurement noise floor

In ETS delay scanning is very fast, thus the acquired signal in the frequency domain is broadband. A complete time window (i.e. over 1/*f*_*pump*_) trace generally corresponds to a frequency band in the radio-frequency (RF) domain from Δ*f* up to ∼*f*_*pump*_/2. The upper signal frequency is generally limited to *f*_*pump*_/2 unless the signal is aliased (i.e. under-sampled by the laser). In our experiments, we have used *f*_*pump*_/Δ*f* ∼ 100,000, which translates fast ultrasonic echoes of ∼100 GHz to 1 MHz, Brillouin oscillations of ∼10 GHz to 100 kHz and slow thermal decays to 1–10 kHz in the RF domain. Therefore, the full ETS signal detection requires considerations of noise contributions over a wide frequency range spanning multiple orders of magnitude.

At high signal frequencies (such as above 300 kHz in the case of our laser) an ultra-low noise floor of a solid-state laser can be exploited. We have shown that shot-noise-limited detection can be obtained even with 15 mW of average power on a high-responsivity photodiode using scheme B [20]. The relative reflectivity in this case is found by dividing the amplified signal voltage by the DC voltage and the calibrated gain of the voltage amplifier. Scheme B does not utilize balanced detection since it is shot-noise limited, so balancing would only increase noise. However, while scheme B offers excellent performance for high frequencies, the bias-tee suppresses the low-frequency signals and hence it does not capture thermal decays. Conversely, scheme A resolves all frequencies, and hence can capture thermal decays, but the data at these lower frequencies are affected by the environmental and laser noise thus reducing sensitivity. Therefore, by digital balancing scheme A at low frequencies and switching to scheme B at high frequencies, we achieve high sensitivity across the whole frequency range. In the case of scheme A, the relative reflectivity is found by calculating the difference between the signal voltage and the scaled reference voltage and dividing the difference by the DC level of the signal. Based on these considerations, we obtain the normalized reflectivity by combining the two measurements in the frequency domain:

(1) $$\frac{\Delta R}{R}=F^{-1}\left[F\left(\frac{V_{\mathrm{signal}}^{A}-s\times V_{\mathrm{reference}}^{A}}{\langle V_{\mathrm{signal}}^{A}\rangle }\right)\times T+F\left(\frac{V_{\mathrm{signal}}^{B}}{g\langle V_{\mathrm{signal}}^{A}\rangle }\right)\times \left(1-T\right)\right].$$

Here $\langle V_{signal}^{A}\rangle$ indicates the DC voltage of the signal, *s* is the scale for the reference voltage such that its DC level matches the DC of the signal, *g* is the gain of the voltage amplifier andF denotes the Fourier transform which we implement in the digital domain by the Fast Fourier Transform. *T* is the digital low-pass filter defining the signal frequency domain stitching which we perform smoothly at 150 kHz. Finally, after determining Δ*R/R* using Eq. (1), we apply a digital low-pass frequency filter of bandwidth *B=* 40 MHz to remove any frequency components which are under-sampled by the laser. This frequency filter in our case defines the measurement time resolution Δ*τ* = 156 fs:

(2) $$\Delta \tau =\frac{\Delta f}{B\times f_{probe}}$$

**Fig. 2:**
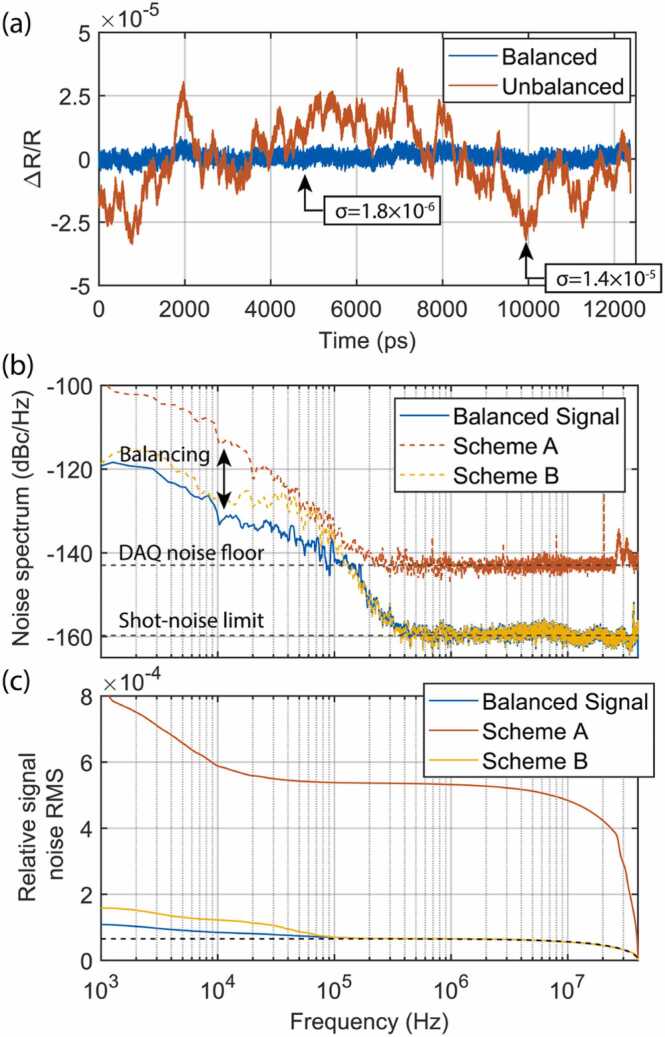
(a) Balanced and unbalanced traces after 4096 averages over the complete 12.5 ns acquisition time window acquired at 500 Hz update rate for a single trace. σ indicates the standard deviation of the entire time trace. (b) Noise spectrum (one-sided power spectral density of the relative signal noise) extracted from the averaged traces and scaled to the noise per single acquisition. The shot-noise limit is estimated based on the experimental parameters. Data acquisition (DAQ) card noise floor is calculated based on its specification. (c) Relative signal noise root-mean-square (RMS) obtained by integrating the data from (b). In the integral for scheme A, the 20 MHz peak (which is an aliased 80 MHz signal) was omitted for clarity purposes. Dashed line in the plot indicates the integrated shot-noise limit.

An example of the balanced and unbalanced traces (with the pump being absent on a sample) is shown in Fig. 2a. The signal balancing reduces the standard deviation over the complete time window nearly by an order of magnitude. Over 4096 averages we obtain 1.8 × 10^−6^ standard deviation on the Δ*R/R* value for the entire 12.5 ns measurement time window. The shot-noise limit can be estimated as described in [20] to be 1 × 10^−6^ indicating a close to shot-noise-limited acquisition condition over the complete RF detection bandwidth. In the experiment, the shot-noise floor is mostly originating from the poor responsivity to the 525 nm light of the available InGaAs detector. It is also important to note that the trace shown is free of any crosstalk, even though the two pulse trains share all the same cavity optics. This condition would not be the case if pulses would overlap on active cavity elements. For example, such overlap is inevitable in single-cavity dual-comb fiber lasers [22].

Next, we study in detail the effectiveness of digital balancing. First, we numerically calculate the one-sided power spectral density of the relative signal noise (noise spectrum later in the text):

(3) $$S\left(f\right)=\frac{2|F\left(V(t)\right)(f){|}^{2}}{\int_{0}^{\infty }|F\left(V(t)\right)(f){|}^{2}df}\times N_{\mathrm{avg}.}$$

Here *V*(*t*) denotes the voltage signal (which has been averaged by the data acquisition card) and *df* indicates integration over the frequency domain. The equation is scaled by the number of averages so that the acquired noise spectrum is representative of a single acquisition. The noise spectrum can be integrated to obtain the relative signal noise root-mean-square (RMS) over a frequency band of interest. We calculate the integral by integrating from high to low frequencies:

(4) $$\sigma_{\mathrm{RMS}}\left(f,f_{\max}\right)=\sqrt{\int_{f}^{f_{\max}}S\left(f\prime \right)df\prime }.$$

The calculated noise spectra and the relative signal noise RMS versus the integration frequency are shown in Fig. 2b and c. The maximum integration frequency *f*_max_ is set to 40 MHz in our case. From Fig. 2b we can see that the reference signal is limited by the detection noise floor for frequencies above 200 kHz. The frequency domain stitching for the unbalanced trace allowed for a much lower noise floor, reaching the calculated shot-noise limit for frequencies above 300 kHz. Finally, the digital balancing successfully suppresses significant technical noise for frequencies below < 150 kHz. From the relative signal noise RMS, it is clear that the residual unbalanced noise has a minimal contribution to the overall noise and the acquisition is mainly shot-noise limited.

Next, we discuss the implications of the shot-noise-limited detection over the complete acquisition bandwidth. As it was discussed in [20], the noise floor in the relative reflectivity trace can be predicted by:

(5) $$\sigma_{\mathrm{shot}-\mathrm{noise}}=\sqrt{\frac{2qB}{P_{\mathrm{avg}}R_{\lambda }}}.$$

Here *q* is the elementary charge, *B* is the signal detection bandwidth, *P*_avg_ is the average power on the photodetector, and *R*_*λ*_ is the photodetector responsivity. Since the shot noise reduces as the square root to the number of averages and thus also scales inversely with the square root of the acquisition time, we can expand the analysis further. By combining this information with Eq. (1) we derive the shot-noise-limited noise floor for different acquisition and laser parameters:

(6) $$\sigma_{\mathrm{shot}-\mathrm{noise}}=\sqrt{\frac{2q}{P_{\mathrm{avg}}R_{\lambda }\Delta \tau f_{\mathrm{probe}}t_{\mathrm{aq}}}}.$$

Here Δ*τ* is the measurement time resolution defined in Eq. (2), and *t*_aq_ is the signal measurement duration. The calculation assumes that the signal is not in an aliased condition and that the acquisition time is an integer multiple of 1/Δ*f*. From this equation, it can be concluded that the optimal measurement performance requires a high average power on the photodetector, a high responsivity photodetector and it does not directly depend on the repetition rate difference. The time resolution will provide a significant trade-off. If limited time resolution is sufficient, very fast signal acquisition can be obtained. For example, if the desired time resolution is restricted to 3 ps, then with an 80 MHz repetition rate laser (12.5 ns delay scan range) one could use 10 kHz repetition rate difference and thus measure 10,000 sample points per second with 3 × 10^−5^ detection sensitivity per pixel (here assuming 15 mW of probe average power and InGaAs detector responsivity of 0.8 A/W at 1050 nm) thanks to the digital signal balancing approach. Thus, near-real-time thermoreflectance imaging is well within reach for such a system.

On the other hand, in the limit of low optical powers, thermal (Johnson) noise may start to dominate. Since the thermal noise can be treated as white noise, we can apply the same analysis as above and predict the noise floor in the case of a thermal-noise-limited acquisition:

(7) $$\sigma_{\mathrm{thermal}-\mathrm{noise}}=\frac{1}{P_{\mathrm{avg}}R_{\lambda }}\sqrt{\frac{4k_{B}T}{R_{\Omega }\Delta \tau f_{\mathrm{probe}}t_{\mathrm{aq}}}}.$$

Here *k*_B_ is the Boltzmann constant, *T* is the detector temperature and *R*_Ω_ is the load resistance. The equation indicates a high noise sensitivity to the probe optical power. Assuming 295 K temperature, 50 Ω load and 0.8 A/W detector responsivity we estimate that for average powers below 1 mW thermal noise starts to dominate the shot noise. For example, consider the case described above. If the probe power were to be reduced by 100 times, i.e., from 15 mW to 150 μW, then the acquisition time, based on the shot-noise considerations alone would need to be increased by 100 times to compensate for the higher noise. However, because the measurement would become thermal-noise limited, the actual acquisition time would need to be increased 800 times. This example demonstrates how it is important to achieve the shot-noise limited acquisition regime. Our presented measurement approach enables achieving shot-noise limit at high average powers without compromises in signal bandwidth due to conveniently employed wide bandwidth voltage amplifier. Such an approach is well-suited also for extending measurement speeds by employing very high repetition rate lasers, such as single-cavity dual-comb oscillators operated at GHz repetition rates [25].

## 4 Thin-film sample measurement

As the next step, we demonstrate the described measurement system for a device under test that is a tungsten thin-film deposited on a thermally oxidized silicon wafer. We chose to study tungsten because it is challenging to measure and yet it is abundant in the microelectronics industry thus demonstrating the relevance of our measurement system. We use the previously described SHG-SHG pump-probe setup. We expose the sample with 2 mJ/cm^2^ of average pump fluence. We collected 4096 averages with 500 Hz Δ*f* setting which corresponds to 8.2 s of integration time. Since our data acquisition card was not capturing every trigger event due to limited processing power, the actual acquisition time was slightly longer. Trigger interruption-free data processing could be implemented for example by using more advanced acquisition cards, especially such that use a graphical processing unit (GPU) or field-programmable gate array (FPGA) to perform real-time processing calculations. The number of averages is chosen to be consistent with the noise analysis discussed in the section above, however, based on the observed noise floor and signal strength, significantly shorter acquisition times could be used to extract the thin-film thickness.

**Fig. 3:**
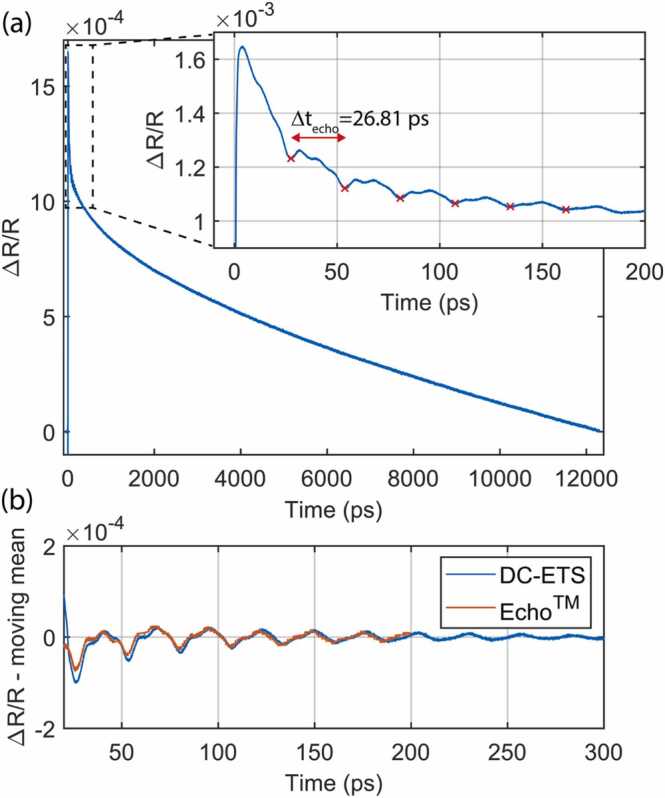
(a) Measured tungsten thin-film response over long and short (zoom-in) time scales. The signal is obtained from 4096 averages with 500 Hz repetition rate difference. The red crosses show the first 6 thin-film echoes spaced by 26.81 ps. (b) Direct comparison between dual-comb ETS and a mechanical delay-based baseline measurement with Echo™ System (Onto Innovation Inc.). Due to different excitation conditions, we have removed slightly different thermal response by subtracting a slowly moving mean and scaling the Echo™ data with a factor of 2.

In Fig. 3a we show the digitally balanced measurement of tungsten thin-film. Slow thermal decay is visible over the complete trace and zooming in around the first 200 ps reveals ultrasonic echoes which are separated by (26.81 ± 0.17) ps. Here, for the uncertainty, we use the 95 % confidence interval of a linear fit on the time of arrival of the first 6 echoes. Considering the longitudinal speed of sound of 5.22 nm/ps [26] as expected for α-phase tungsten [27] we can estimate the tungsten layer thickness to be (700 ± 4) Å. This result was verified by a mechanical delay line-based measurement of the same sample with a Echo™ System (Onto Innovation Inc.) which determined 694 Å thin-film thickness with a typical 1 % accuracy [28] indicating great agreement between the two measurement systems (Fig. 3b). The exact measurement position on the wafer was not controlled, and thus wafer non-uniformity could also have contributed to the small difference between the two measurements.

## 5 Conclusions

Practical deployment of ultrafast photoacoustic techniques requires simple, compact and high-performance measurement systems. In this letter, we demonstrate an appealing practical implementation to efficient pump-probe long delay range sampling using a single-cavity dual-comb laser. The single-cavity laser serves as a complete ETS (also referred to as ASOPS) system with higher timing precision and ultra-low intensity noise which helps to construct an efficient pump-probe measurement apparatus. In our current implementation, the measurement of Δ*f* and *f*_pump_ used two separate devices. For future work, we plan to measure both using a common reference clock so that the ratio Δ*f*/*f*_pump_ is not sensitive to the accuracy of the clock. We have analyzed contributing noise factors in ETS measurements, and we have identified a high-performance measurement scheme which allows obtaining shot-noise-limited measurements even at high average power on a photodetector. In addition, we have demonstrated digital signal balancing, which is compatible with a very rapid, single-trace per pixel-type signal acquisition necessary for high-performance pump-probe microscopy deployment. Finally, we demonstrate a picosecond ultrasonics measurement on a tungsten thin film where we obtain precise layer thickness identification of (700 ± 4) Å well matching to an independent characterization using an Echo™ System (Onto Innovation Inc.).

## Declaration of Competing Interest

The authors declare the following financial interests/personal relationships which may be considered as potential competing interests: J. Pupeikis, B. Willenberg, C. R. Phillips and U. Keller reports financial support was provided by Swiss National Science Foundation. M. Mehendele and G. A. Antonelli reports a relationship with Onto Innovation that includes: employment.

## Data Availability

Data will be made available on request.

## References

[1] Maiuri M., Garavelli M., Cerullo G. (2020). Ultrafast spectroscopy: state of the art and open challenges. J. Am. Chem. Soc..

[2] Thomsen C., Strait J., Vardeny Z., Maris H.J., Tauc J., Hauser J.J. (1984). Coherent phonon generation and detection by picosecond light pulses. Phys. Rev. Lett..

[3] Maris H. (1997). Brief pulses of high-frequency sound allow experimenters to probe connections inside a computer chip. Sci. Am..

[4] Dehoux T., Ghanem M.A., Zouani O.F., Rampnoux J.-M., Guillet Y., Dilhaire S., Durrieu M.-C., Audoin B. (2015). All-optical broadband ultrasonography of single cells. Sci. Rep..

[5] Liu L., Plawinski L., Durrieu M.-C., Audoin B. (2019). Label-free multi-parametric imaging of single cells: dual picosecond optoacoustic microscopy. J. Biophotonics.

[6] Pérez-Cota F., Fuentes-Domínguez R., La Cavera S., Hardiman W., Yao M., Setchfield K., Moradi E., Naznin S., Wright A., Webb K.F., Huett A., Friel C., Sottile V., Elsheikha H.M., Smith R.J., Clark M. (2020). Picosecond ultrasonics for elasticity-based imaging and characterization of biological cells. J. Appl. Phys..

[7] Jiang P., Qian X., Yang R. (2018). Tutorial: time-domain thermoreflectance (TDTR) for thermal property characterization of bulk and thin film materials. J. Appl. Phys..

[8] Capinski W.S., Maris H.J. (1996). Improved apparatus for picosecond pump‐and‐probe optical measurements. Rev. Sci. Instrum..

[9] Weingarten K.J., Rodwell M.J.W., Heinrich H.K., Kolner B.H., Bloom D.M. (1985). Direct electro-optic sampling of GaAs integrated circuits. Electron. Lett..

[10] Elzinga P.A., Kneisler R.J., Lytle F.E., Jiang Y., King G.B., Laurendeau N.M. (1987). Pump/probe method for fast analysis of visible spectral signatures utilizing asynchronous optical sampling. Appl. Opt..

[11] Kliebisch O., Heinecke D.C., Dekorsy T. (2016). Ultrafast time-domain spectroscopy system using 10 GHz asynchronous optical sampling with 100 kHz scan rate. Opt. Express.

[12] Dekorsy T., Hudert F., Cerna R., Schäfer H., Janke C., Bartels A., Köhler K., Braun S., Wiemer M., Mantl S. (2 2006). Coherent acoustic phonons in nanostructures investigated by asynchronous optical sampling. Proc. SPIE 6393, Nanophotonics for Communication: Materials, Devices, and Systems.

[13] Stoica V.A., Sheu Y.-M., Reis D.A., Clarke R. (2008). Wideband detection of transient solid-state dynamics using ultrafast fiber lasers and asynchronous optical sampling. Opt. Express.

[14] Abbas A., Guillet Y., Rampnoux J.-M., Rigail P., Mottay E., Audoin B., Dilhaire S. (2014). Picosecond time resolved opto-acoustic imaging with 48 MHz frequency resolution. Opt. Express.

[15] Mehravar S., Norwood R.A., Peyghambarian N., Kieu K. (2016). Real-time dual-comb spectroscopy with a free-running bidirectionally mode-locked fiber laser. Appl. Phys. Lett..

[16] Link S.M., Klenner A., Mangold M., Zaugg C.A., Golling M., Tilma B.W., Keller U. (2015). Dual-comb modelocked laser. Opt. Express.

[17] Ideguchi T., Nakamura T., Kobayashi Y., Goda K. (2016). Kerr-lens mode-locked bidirectional dual-comb ring laser for broadband dual-comb spectroscopy. Optica.

[18] Endo M., Shoji T.D., Schibli T.R. (2018). Ultralow noise optical frequency combs. IEEE J. Sel. Top. Quantum Electron.

[19] Pupeikis J., Willenberg B., Camenzind S.L., Benayad A., Camy P., Phillips C.R., Keller U. (2022). Spatially multiplexed single-cavity dual-comb laser. Optica.

[20] Pupeikis J., Willenberg B., Bruno F., Hettich M., Nussbaum-Lapping A., Golling M., Bauer C.P., Camenzind S.L., Benayad A., Camy P., Audoin B., Phillips C.R., Keller U. (2021). Picosecond ultrasonics with a free-running dual-comb laser. Opt. Express.

[21] Modsching N., Drs J., Brochard P., Fischer J., Schilt S., Wittwer V.J., Südmeyer T. (2021). High-power dual-comb thin-disk laser oscillator for fast high-resolution spectroscopy. Opt. Express.

[22] Fellinger J., Fellinger J., Mayer A.S., Winkler G., Grosinger W., Truong G.-W., Droste S., Li C., Heyl C.M., Heyl C.M., Hartl I., Heckl O.H., Heckl O.H. (2019). Tunable dual-comb from an all-polarization-maintaining single-cavity dual-color Yb:fiber laser. Opt. Express.

[23] Nussbaum-Lapping A., Phillips C.R., Willenberg B., Pupeikis J., Keller U. (2022). Absolute SESAM characterization via polarization-resolved non-collinear equivalent time sampling. Appl. Phys. B.

[24] H.J. Maris, R.J. Stoner, 2002. Optical stress generator and detector, United States patent US6400449B2 (June 4, 2002).

[25] C.R. Phillips, B. Willenberg, A. Nussbaum-Lapping, F. Callegari, S.L. Camenzind, J. Pupeikis, U. Keller, Coherently averaged dual-comb spectroscopy with a low-noise and high-power free-running gigahertz dual-comb laser, arXiv:2211.01368 (2022) https://arxiv.org/abs/2211.01368. 10.1364/OE.479356 36859848

[26] Featherston F.H., Neighbours J.R. (1963). Elastic constants of tantalum, tungsten, and molybdenum. Phys. Rev..

[27] Lee J.-S., Cho J., You C.-Y. (2016). Growth and characterization of α and β-phase tungsten films on various substrates. J. Vac. Sci. Technol. A.

[28] Comprehensive In-Line Metrology for Advanced RDL Process Monitoring - Onto Innovation, 〈https://ontoinnovation.com/library/comprehensive-in-line-metrology-for-advanced-rdl-process-monitoring〉.

